# Nonanatomic versus anatomic techniques in spring ligament reconstruction: biomechanical assessment via a finite element model

**DOI:** 10.1186/s13018-019-1154-5

**Published:** 2019-04-29

**Authors:** Can Xu, Ming qing Li, Chenggong Wang, Hua Liu

**Affiliations:** Department of Orthopaedics, Xiangya Hospital, Central South University, Changsha, 410008 China

**Keywords:** Finite element, Flatfoot, Spring ligament reconstruction, Nonanatomic, Contact characteristics

## Abstract

**Background:**

Several approaches to spring ligament reconstruction have been reported. However, a comparative study of nonanatomic and anatomic techniques with respect to biomechanical responses, such as kinematics and contact characteristics, has not been previously performed via a finite element analysis. The purpose of this study was to evaluate the biomechanical results of such spring ligament reconstructions via a finite element analysis.

**Methods:**

A three-dimensional finite element model of the foot was developed and validated, and four reconstruction methods were simulated. The talonavicular dorsiflexion and abduction, hindfoot valgus, and contact characteristics in the Chopart joints were quantified in each model.

**Results:**

Nonanatomic reconstructions corrected the talonavicular and hindfoot deformities to a greater extent than the anatomic reconstructions. The anatomic techniques also corrected the abduction and dorsiflexion deformities, although they presented insufficient power to correct for hindfoot valgus. None of the procedures restored the contact characteristics of the talonavicular and calcaneocuboid joints to those of a normal condition.

**Conclusion:**

Nonanatomic reconstruction of the spring ligament complex provided the greatest correction for midfoot and hindfoot misalignments in flatfoot. Severe deformities with large amounts of midfoot pronation and hindfoot valgus may be better treated with nonanatomic reconstruction methods. The spring ligament reconstruction method may mitigate the need for nonanatomic bony procedures associated with complications and allows for the preservation of the triple joint complex.

## Background

The spring ligament is the main static supporter of the medial longitudinal arch, which functions as a sling to support the talar head and prevent it from falling into plantarflexion and adduction [1]. This ligament is classically described by two bundles, the superomedial calcaneonavicular (SMCN) ligament and the inferior calcaneonavicular (ICN) ligament. The ICN ligament is a small fibrous structure that lies directly beneath the talonavicular joint, whereas the SMCN ligament is a thicker band partly composed of fibrocartilage, and it is located directly medial to the posterior tibial tendon insertion and blends into the deltoid complex [2]. Besides these 2 components, a “third ligamentt” that runs from the notch between the anterior and middle calcaneal articular facets to the navicular tuberosity is identified by Taniguchi et al. [3]. However, it is not always present and difficult to recognize [4].

The spring ligament works in accordance with dynamic structures, including the posterior tibial tendon, to support the head of the talus. In patients with adult-acquired flatfoot deformity (AAFD), the posterior tibial tendon and spring ligament are often deficient, resulting in peritalar subluxation and valgus heel malalignment [2, 5–7]. Many operations are available for correction of a flexible AAFD, such as several joint-preserving osteotomies, tendon transfers, and soft tissue reconstructive techniques [8]. However, the nonanatomic procedures like sliding calcaneal osteotomy, lateral column lengthening, and flexor digitorum longus (FDL) transfer could not reconstitute the plantar arch in a satisfactory way [2]. Reconstruction of the spring ligament with robust graft tissue is a useful method to resist the strain at the talonavicular joint and maintain correction. When used as an adjunct procedure in flatfoot treatment, the reconstruction of spring ligament may obviate the need of nonanatomic procedures like lateral column lengthening and even subtalar and midfoot fusions in severe deformities [9].

Multiple techniques for spring ligament reconstruction, both anatomic and nonanatomic, have been described, mostly on cadaveric model [5, 6, 10–12]. Baxter and colleagues compared 3 different reconstruction techniques with peroneus longus tendon on a cadaveric model [5]. Choi and colleagues proposed a procedure to spring ligament reconstruction that also used the peroneus longus tendon and compared 3 reconstructive methods on a cadaveric model [6]. Thordarson and colleagues compared the use of peroneus longus, tibialis anterior, and Achilles tendon transfers on a cadaveric model [13]. Besides the cadaveric studies, several techniques were also described on clinical series [12, 14, 15]. However, the patients commonly received concomitant procedures like FDL transfer, sliding calcaneal osteotomy, or first tarsometatarsal fusion. Thus, the biomechanical response of the spring ligament reconstruction was difficult to measure. To our knowledge, a comparative study of nonanatomic and anatomic techniques without concomitant procedures via a finite element analysis has not been previously performed.

In our study, a three-dimensional foot finite element (FE) model was developed to compare the biomechanical behaviors of the anatomic and nonanatomic reconstructions for flatfoot treatment. We sought to determine how different spring ligament reconstructions affect biomechanical conditions in a model of flatfoot deformity. Specifically, we tested 4 reconstruction techniques that aimed to recreate different portions of the ligaments that support the medial talonavicular joint with the goal of providing recommendations for the optimal design of spring ligament reconstruction for AAFD.

## Methods

**Fig. 1:**
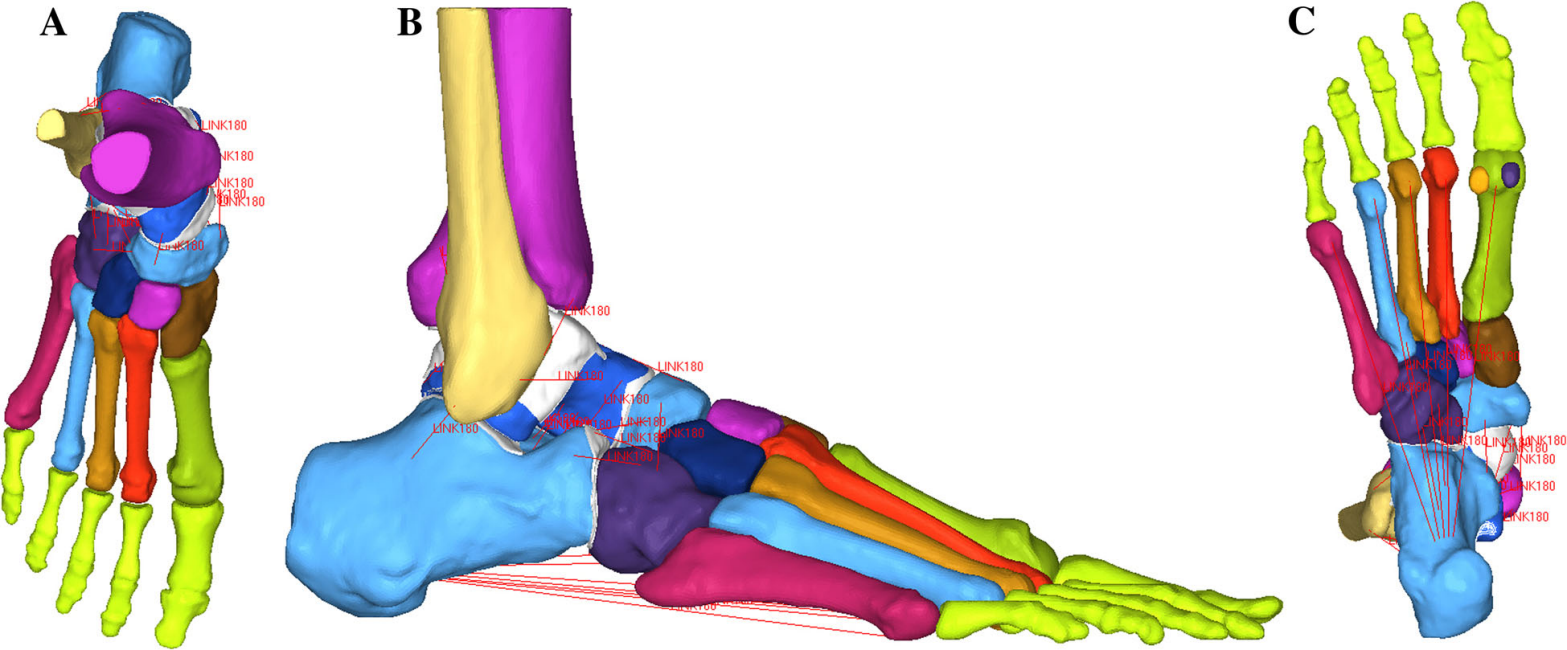
The FE model: **a** dorsal view, **b** lateral view, and **c** planter view

Computerized tomography (CT) and magnetic resonance imaging (MRI) scans of a healthy male volunteer with intact bone structures (30-years old; 170 cm in height; 68 kg in weight) were used to develop the bony and soft tissue geometry, respectively. The CT images were segmented to obtain the boundaries of each bone using Mimics 17.0 software (Materialise, Belgium). The skeletal components were processed using Geomagic Studio 2013 (Geomagic, USA). The whole model included 28 bones (tibia, fibula and 26 foot bones), sesamoids, plantar fascia, 24 main ligaments (list in Table 1, 2, 3), cartilage, and soft tissue. The forefoot ligaments were simplified for these ligaments may not apply to the evaluation of the flatfoot reconstruction. All components were then imported and assembled in the FE package (ABAQUS 6.13, SIMULIA Inc., US). The bones were meshed with tetrahedral elements. All bones were idealized as homogeneous, isotropic material and assigned rigid properties (Fig. 1).

**Table 1:** Tibia, fibula, and hindfoot ligament properties [20, 21]

Ligament	*a* (N)	*b*
Anterior talofibular	7.18	12.50
Anterior tibiofibular	5.52	22.63
Anterior tibiotalar	2.06	20.11
Calcaneofibular	0.20	49.63
Posterior talofibular	0.14	44.35
Posterior tibiofibular	6.87	20.07
Posterior tibiotalar	1.34	28.65
Tibiocalcaneal	0.51	45.99
Tibionavicular	*k* = 39.1 N/mm [21]

**Table 2:** Calcaneus, talus and midfoot bone ligament properties [22, 23]

Ligament	Area (mm^2^)	Area ratio
Anterior talocalcaneal	14.4	0.229
Posterior talocalcaneal	14.96	0.238
Lateral talocalcaneal	6.84	0.109
Medial talocalcaneal	14.91	0.237
Interosseous talocalcaneal	72.80	1.158
Dorsal talonavicular	35.15	0.559
Interosseous calcaneocuboid	72.80	1.158
Plantar calcaneocuboid	98.70	1.570
Inferior calcaneonavicular	9.23	0.147
Superomedial calcaneonavicular	161.00	2.560
Dorsal cuboideonavicular	13.10	0.208
Plantar cuboideonavicular	27.80	0.442
Interosseous cuboideonavicular	14.01	0.223

**Table 3:** Plantar fascia and long/short plantar ligament properties [24, 25]

Ligament	Stiffness *k* (N/mm)
Plantar fascia	203.3 [24]
Long/short plantar ligament	75.9 [25]

Ligaments were developed manually in 3D reconstruction models based on MRI. Insertion locations were determined by inspection of anatomy as presented in the MRI scans and in consultation with previous dissections documented in journal papers and textbooks [16, 17]. Force in each ligament was generated as a function of the ligament stiffness and in situ strain. Ligament stiffness was assigned to tension-only structures [18, 19]. The tibial and fibular ligaments were assigned non-linear force-displacement equations [20], whereas the tibionavicular ligament was assigned a linear stiffness [21]. The material properties of the midfoot ligaments and hindfoot ligaments connecting the talus and calcaneus were assumed to be equivalent to those of the anterior talofibular ligament scaled by the ratio between their respective cross-sectional areas [22–25] (Tables 1, 2 and 3). In situ strain was assigned for the ligaments with values published in previous literature and a 4% in situ strain was applied to other ligament element without published values [18, 19, 26–30].

The ligament properties for eight ligaments that connect the tibia and fibula with the bones of the foot and ankle are expressed as curve fit data (a and b) for an elastic force-strain response function [20] (*T*(*ε*) = *a*(*e*^bε^ − 1)). The stiffness, *k*, of the tibionavicular ligament is also provided [21].

The properties of the calcaneal, talar and midfoot bone ligaments were assumed to be equivalent to those of the anterior talofibular ligament and scaled by their relative cross-sectional areas. The anterior talofibular ligament has a cross-sectional area of 62.85 mm^2^ [23], and the areas for all other ligaments were provided by Mkandawire [23] and Shin [22].

The linear elastic stiffness values reported in the literature for the long/short plantar ligaments and plantar fascia are provided [24, 25].

Cartilage thicknesses of the tibiotalar*, subtalar,* talonavicular, and calcaneocuboid joints cartilage were measured via T1-weighted MRI. Then, the cartilage was added to these joints. *The* cartilage was defined *as being neo-Hookean hyperelastic with E = 10 MPa and ν = 0.45* [31]*. Surface-to-surface contact* elements were introduced *bet*ween cartilages with a coefficient of friction (*μ*) of 0 to simulate the relative articulating movement. The peroneus longus tendon used as grafts was considered a homogeneous isotropic linear elastic material. The initial cross-sectional areas were considered 37 mm^2^, and the elastic modulus values were 2769 MP [32].

**Fig. 2:**
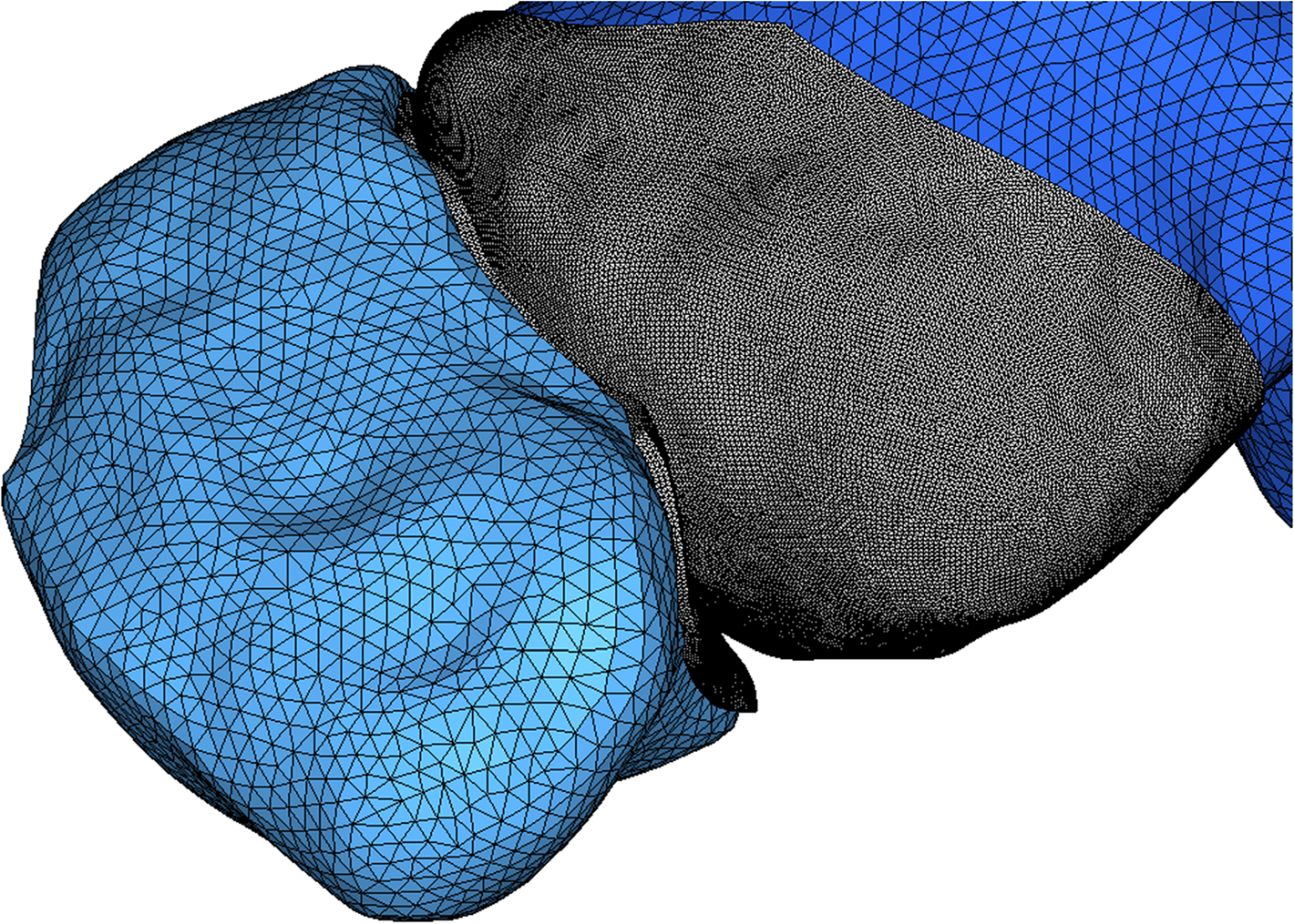
A close-up view of the mesh for talonavicular joint

To achieve a reasonable mesh size without compromising the calculation time, the trial-error approach was employed in mesh convergence study [33]. For all models, the bones were meshed with a mean element volume of 1.5 mm^3^, and the cartilage was meshed with a mean element volume of 0.2 mm^3^; these values were deemed adequate based on the results of mesh convergence study. The entire model consisted of 178,370 elements and 269,517 nodes (Figs. 1 and 2)

### Simulated conditions

**Fig. 3:**
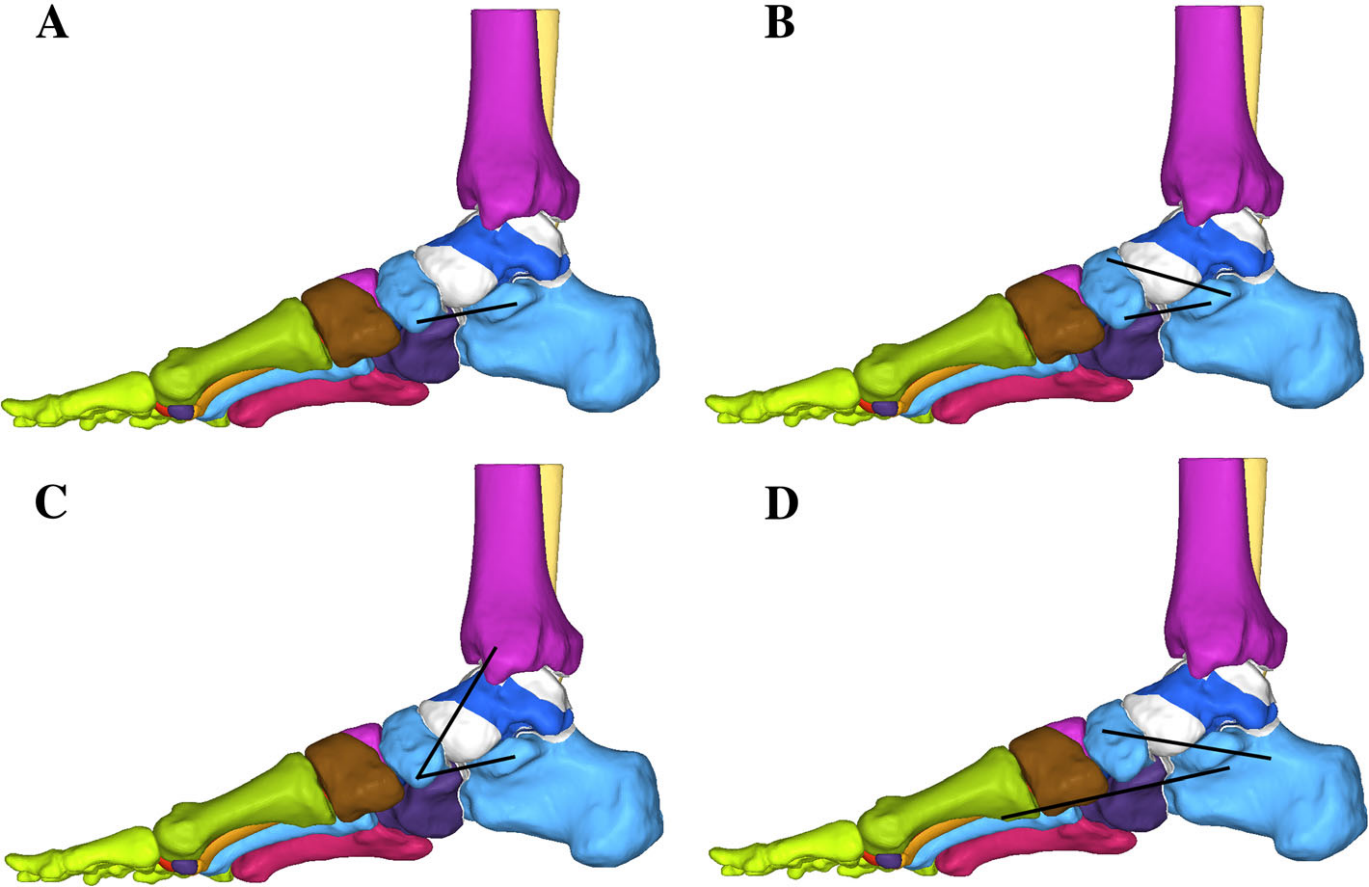
Four models with reconstructed tendon grafts: inferior spring ligament reconstruction (**a**), anatomic spring ligament reconstruction (**b**), combined superomedial/inferior ligament reconstruction (**c**), and combined superomedial/plantar ligament reconstruction (**d**)

Four reconstruction methods were simulated. The peroneus longus tendon was used as the graft. Some parts of the tendon graft pass through bone tunnel; however, this is not simulated in our model. Method a/b included anatomic reconstructions, and method c/d included the following nonanatomic reconstructions:

- Inferior spring ligament reconstruction (ISLR): The graft attachment points were determined based on clinical approaches [12]. The graft extended from the hole in the navicular to the calcaneus. This reconstruction technique was aimed to recreate the action of the ICN ligament (Fig. 3a).
- Anatomic spring ligament reconstruction (ASLR): The model was developed based on clinical approaches [5]. The graft was placed from the hole in the anterior calcaneus to the plantar navicular and then from the hole in the dorsal navicular to the posterior calcaneus. This reconstruction technique was performed to recreate the combined action of the ICN and SMCN ligaments and approximated the lines of action of the spring ligament complex (Fig. 3b).
- Combined superomedial/inferior ligament reconstruction (CSILR): The graft attachment points were determined based on clinical approaches [34]. The first part of the graft extended from the hole in the navicular to the calcaneus, and the second part extended from the hole in the navicular to the tibia. This reconstruction was performed to recreate the combined function of the ICN ligament and anterior deltoid ligaments because the anterior deltoid ligament (tibiospring ligament) sometimes blended in with the SMCN ligament [16, 35]. Therefore, the anterior deltoid ligament can also be attenuated in flatfoot [36], and these structures can be reconstructed together (Fig. 3c).
- Combined superomedial/plantar ligament reconstruction (CSPLR): The graft attachment points were determined based on clinical approaches [6]. The peroneus longus tendon was first passed through the anterior calcaneal hole medially to laterally, then passed back through the posterior calcaneal hole laterally to medially, and finally, pulled through the navicular hole from the dorsal to plantar positions. This reconstruction was performed to recreate the combined function of the SMCN ligament and other plantar support structures of the medial longitudinal arch (Fig. 3d).

### Boundary conditions

The model was positioned against a rigid base to mimic a single-leg stance with the foot in the neutral position. The model was loaded axially under the patients’ body weight (680 N) and vertically via the shaft of the tibia and fibula. Linear elements representing the muscle and tendon forces were applied throughout the foot and ankle complex providing further support to the anatomical bony constraints. Muscle tendon activation values were assigned from the literature [18, 19]. To simulate AAFD, the stiffness values of certain ligament properties, including the spring, deltoid, short plantar, and long plantar ligaments and plantar fascia, were reduced by half according to the reported data [19].

### Foot finite element model validation

The model was validated by comparing the anatomical measurement under different loading conditions. The talonavicular dorsiflexion (lateral Meary’s angle) and abduction (anteroposterior Meary’s angle), the hindfoot valgus, and arch height were calculated under body weight. These values were compared with the results from the experiment. Cheung et al. [37] performed a pure vertical compression test in six cadaveric feet, and the average of the six curves reported was used for comparison. Tao et al. [38] measured the vertical deformation of the foot in vivo under gradually increasing loads using a motion capture system. The model was axially loaded from 0 to 600 N in 100-N step increments, and the vertical displacement of the navicular bone was compared with both experimental measurements [37, 38].

### Joint kinematics

**Fig. 4:**
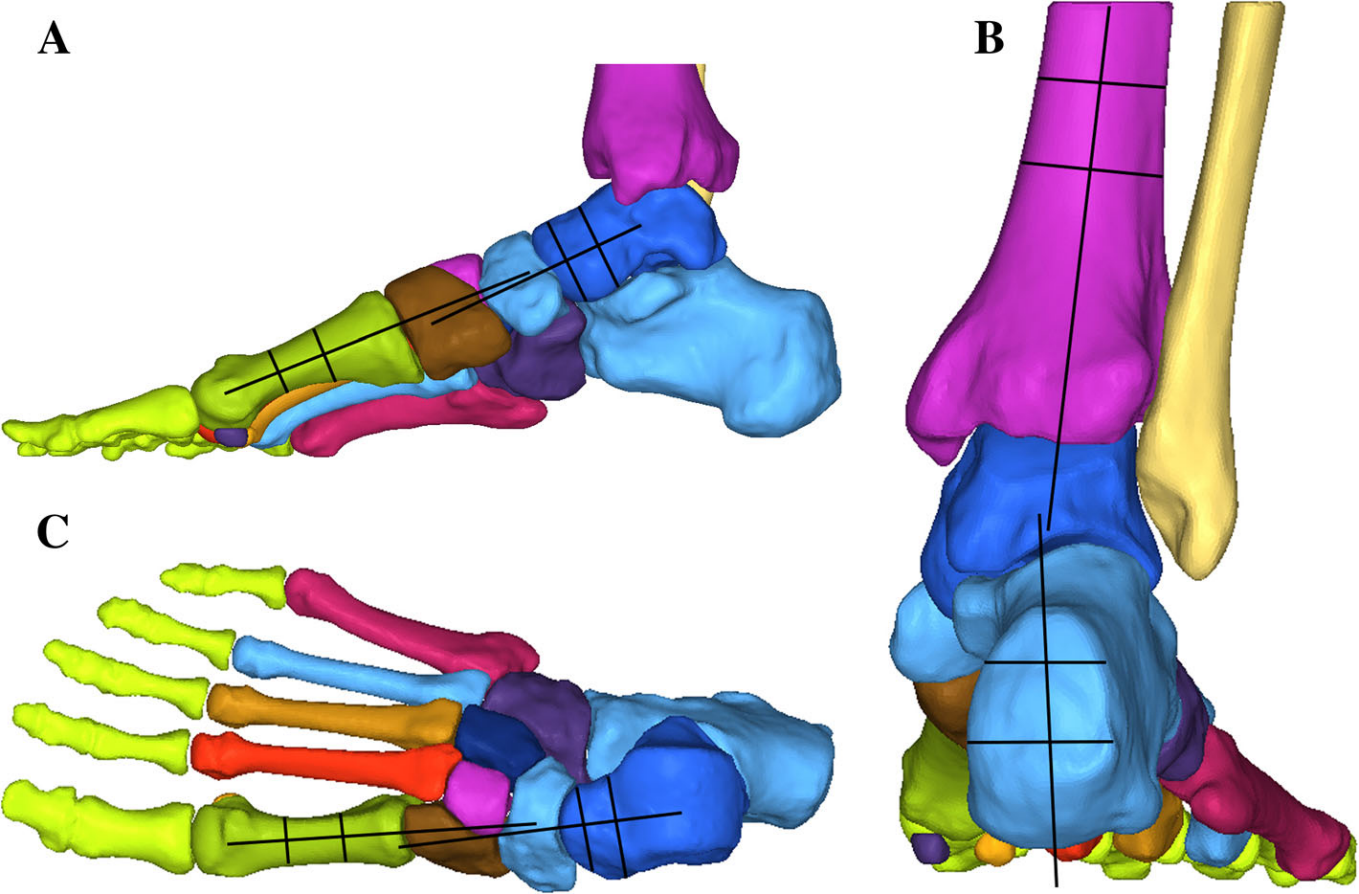
Radiographic measurements: **a** lateral view of foot and ankle showing Meary’s angle, **b** anteroposterior view of foot showing AP Meary’s angle, and **c** posterior view of foot and ankle showing hindfoot valgus

After applying the patient’s body weight, the model was allowed to equilibrate to its final position. Three angular measurements were performed to replicate the clinical radiographic measures (Fig. 4). In lateral view of the foot, Meary’s angle was observed, whereas in anteroposterior (AP) view of the foot, AP Meary’s angle was observed. In posterior view of the ankle, hindfoot (tibiocalcaneal) valgus was observed. The radiographic measurements were identified independently by three investigators and averaged to minimize potential bias. All joint angles measured during the intact condition were considered zero.

### Mean/peak contact pressure and area

The contact area and pressure between talonavicular joint were analyzed under body weight. The resultant contact force can be determined for any joint within the foot/ankle complex. However, focus was placed on the forces generated at the talonavicular articulation to determine the shift in force after the implementation of these procedures.

## Results

### Validation

**Fig. 5:**
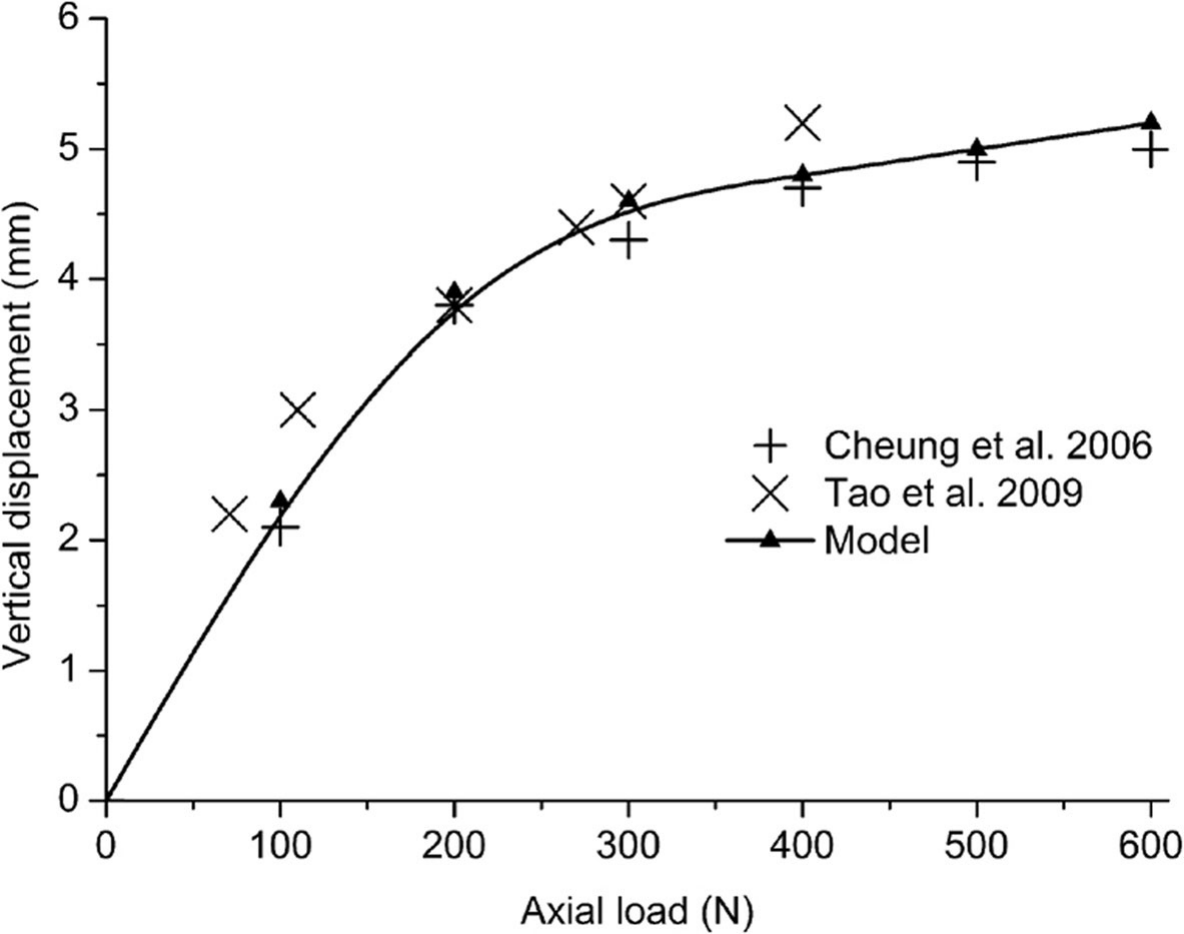
Comparison of the vertical displacement measured by Cheung et al. [37] and Tao et al. [38] with the vertical displacement calculated by the model

The flatfoot model produced a realistic deformity that was consistent with other biomechanical investigations. The node at the tuberosity of the navicular bone in the medial side closest to the planter plane was selected, which is normally used as the reference point in manual measurements. The model accurately reproduced the vertical displacement of the foot under compression and predicted a non-linear response of the foot-ankle complex (i.e., becoming stiffer by increasing the axial load) (Fig. 5).

AP Meary’s angle in cadaveric studies ranges from 4° to 14°, whereas the lateral Meary’s angle spans from 3° to 11° [39–41]. These deformities in our simulation were 7.1° and 4.9°, respectively. Hindfoot valgus was reported in previous cadaveric models from a range of 2° to 6° [39, 41], whereas the valgus deformities in our investigation were 4.7°.

### Joint kinematics

Nonanatomic reconstructions corrected the talonavicular and hindfoot deformities to a greater extent compared with the anatomic reconstructions (Table 4). Although the AP and lateral Mearyla angle of the talonavicular joint were corrected by the ASLR and ISLR techniques, the CSILR and CSPLR methods corrected twice as much of the AP Mearye angle compared with the ISLR. The CSILR and CSPLR methods also corrected 4 times as much of the hindfoot valgus deformity compared with the ISLR, which resulted in a varus hindfoot. The ISLR method did not have a large effect on the hindfoot correction, whereas the other techniques did have a large effect. The CSPLR method had a similar correcting power as the CSILR method, which provided the greatest amount of correction.

**Table 4:** Correction angels of the 4 reconstructions

Reconstruction	Talonavicular deformity (degrees)		Hindfoot deformity (degrees)
	Abduction (AP Meary’s angle)	Dorsiflexion (lateral Meary’s angle)	Valgus
Method ISLR	6.3	4.4	1.5
Method ASLR	8.9	6.5	3.3
Method CSILR	13.7	11.1	7.1
Method CSPLR	12.3	9.8	6.2

### Joint contact

**Fig. 6:**
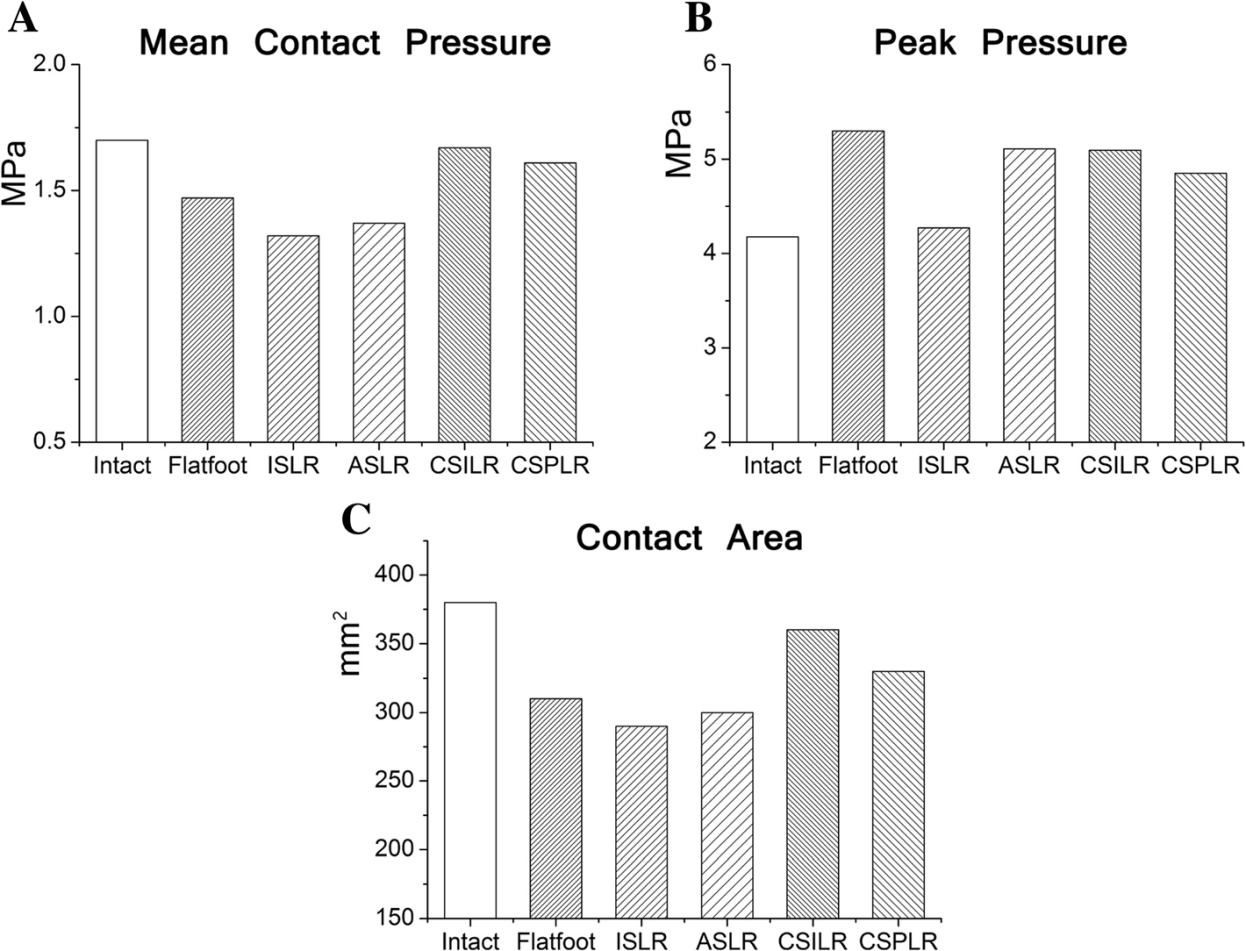
Images of intra-articular talonavicular pressure distributions under body weight for each condition. Pressure is represented in MPa. No reconstruction technique was able to restore the normal joint contact mechanics

The mean contact pressure across the talonavicular joint decreased after flatfoot was modeled, and the force at the calcaneocuboid joint increased for the flatfoot model compared with the normal values. All procedures resulted in a reduction of force across the calcaneocuboid joint surfaces. After implementing the CSILR procedure, the contact pressure across the talonavicular joint increased to a greater degree compared with the intact condition and the other reconstructed conditions (Fig. 6a).

The maximum stress of the talonavicular joint with respect to different procedures was shown in Figure 6b. All reconstruction techniques were able to decrease the peak pressure from the flatfoot condition. The maximum stress on the talonavicular joint was highest in the ASLR procedure, followed by the CSILR, CSPLR, and ISLR procedure.

The contact area across the talonavicular joint in the flatfoot model decreased from the intact condition. For the talonavicular joint, all procedures increased the contact area from the flatfoot condition. The contact area in the talonavicular joint increased to the greatest degree from the flatfoot condition after the CSILR procedure (Fig. 6c).

**Fig. 7:**
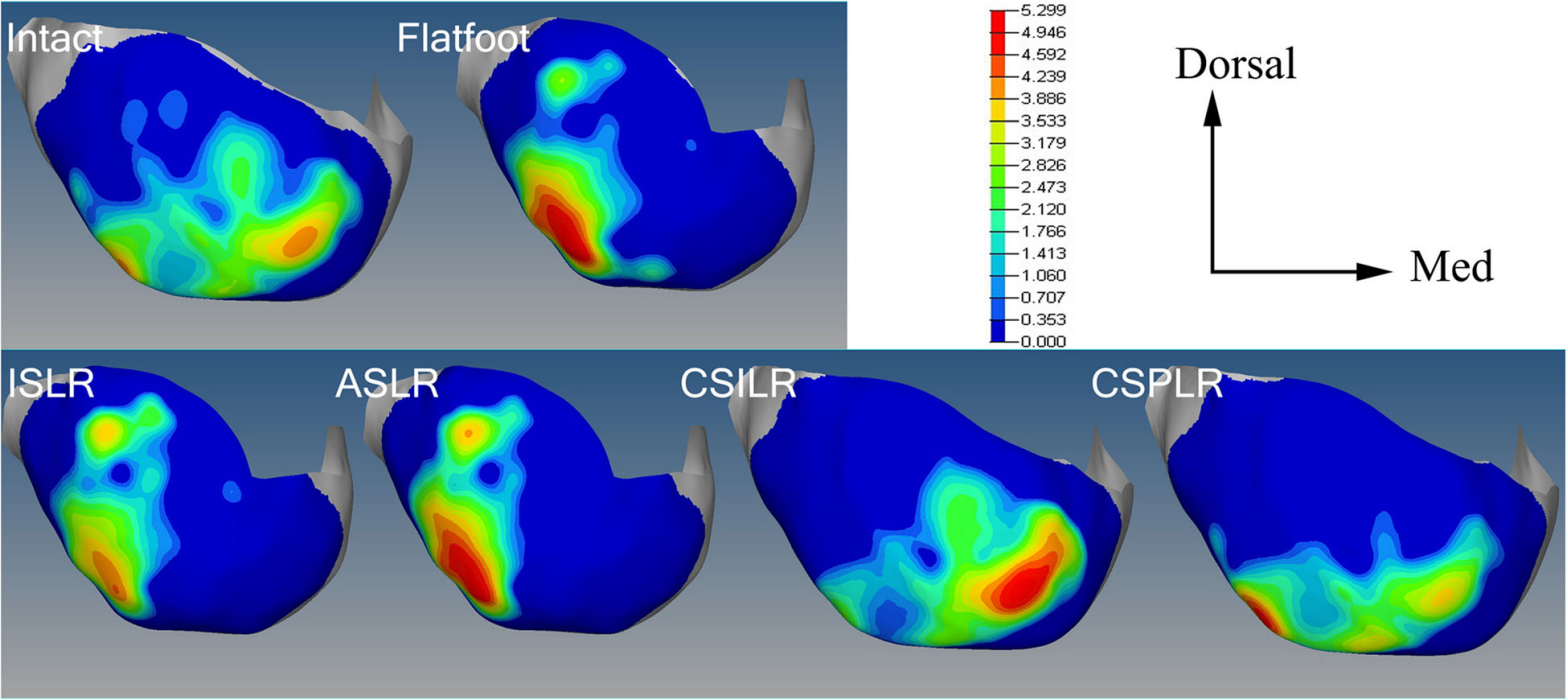
**a** Mean contact pressure, **b** peak pressure, and **c** contact area calculated by FE analysis under body weight for each condition

Figure 7 shows the stress distribution of the talonavicular joint with respect to different procedures. For intact condition, 2 centers of pressure form in the plantar-lateral and medial-dorsal regions of the joint space, with the greatest magnitude of pressure in the medial-dorsal joint. For flatfoot, the pressure is concentrated in the plantar-lateral region. No reconstruction technique was able to restore the normal joint contact mechanics. The stress distribution patterns were similar in flatfoot and anatomic procedures. The contact stress was more concentrated on medial region in the nonanatomic reconstructions in comparison to the anatomic reconstructions.

## Discussion

Spring ligament failure is usually caused by the repetitive stresses of a flatfoot, which causes increased strain on the medial ligament of the foot. Failure of this ligament could lead to progressive subluxation at the talonavicular joint, which may eventually cause enough deformity in the triple joint complex to result in lateral impingement and pain in the hindfoot. Due to the degenerative process that the spring ligament suffers as the AAFD occurs, the native tissue are not suitable for repair. Therefore, spring ligament reconstruction with robust tendon theoretically better withstand the strain at the talonavicular joint and maintain correction of the deformity.

We used a FE model to investigate the effects of anatomic and nonanatomic spring ligament reconstructions in flatfoot treatment. We observed that the nonanatomic reconstructions showed a greater correction of the talonavicular and hindfoot deformities created in our flatfoot model than the anatomic reconstructions. The anatomic techniques also corrected the abduction and dorsiflexion deformities, although their correcting power for hindfoot valgus was insufficient. None of the procedures restored the contact characteristics of the talonavicular and calcaneocuboid joints to a normal condition. The CSILR procedure resulted in peak talonavicular pressures that were most similar to the normal state.

Several cadaveric studies have compared different reconstruction methods of spring ligament. For concomitant procedures that are commonly performed at the time of spring ligament reconstruction in flatfoot surgery, it is difficult to determine the effectiveness of the spring ligament reconstruction. The cadaveric studies provide the opportunity to investigate the real effect of isolated spring ligament reconstruction. Choi et al. [6] compared multiple different techniques of spring ligament reconstruction using the peroneus longus tendon without concomitant procedures. In this study, the most successful pattern was the CSPLR technique, which closely reconstructed the superior medial calcaneonavicular and inferior calcaneonavicular ligaments. Thordarson et al. [13] compared four different techniques for flatfoot reconstruction. In this study, the peroneus longus tendon was transected proximal to the superior peroneal retinaculum. Then, it was delivered through an incision on the medial side of the foot and subsequently passed medial to lateral through a bone tunnel in the calcaneus. Lastly, the end of peroneus longus tendon was secured with a screw on the calcaneus. When compared with the other reconstruction methods, this nonanatomic reconstruction performed better on correction of the deformity. Baxter et al. [5] tested several reconstruction methods by fixing the peroneus longus tendon graft in different positions to recreate the various static soft tissue restraints of the medial longitudinal arch. They also reported that the nonanatomic ligamentous reconstruction of the medial longitudinal arch resulted in a greater correction of the deformity than did the ASLR technique. In our study, the predicted results were similar to those of published data. The CSILR and CSPLR techniques were more powerful in correcting the deformities of flatfoot than were the ASLR and ISLR methods. These findings suggest that anatomic reconstruction of the spring ligament alone may not provide enough talonavicular stability and hindfoot valgus correction.

We observed that the correcting power of reconstruction was closely related to the truss mechanism. Hicks [42] originally described the foot and its ligaments as an arch-like triangular structure or truss. According to the truss mechanism, the hindfoot represents the posterior strut and the forefoot up to the metatarsal heads represents the anterior strut, with both subjected to compression, and the plantar soft structures act as a tie-rod by taking up the tension and eliminating bending [43]. The plantar static soft tissue restraints of the medial longitudinal arch include the spring ligament complex, superficial deltoid ligament, medial talonavicular joint capsule, plantar fascia, and talocalcaneal ligaments [44]. Although the spring ligament complex has been the focus of ligament reconstruction in flatfoot surgery, numerous authors have reported that arch stability is provided by the cumulative effect of several soft tissue restraints [45–47]. In a cadaveric study, Huang et al. observed that the plantar fascia was the most important structure tested, and provided approximately 25% of the medial longitudinal arch stiffness [48]. Reeck et al. [47] reported that the superomedial calcaneonavicular ligament only supports 10% of ground reactive forces transferred through the acetabulum pedis.

The anatomic technique aimed to recreate the function of the ICN and SMCN portions of the spring ligament. Because of the anatomical locations, the ICN portion mainly prevents talonavicular dorsiflexion and the SMCN portion mainly prevents talonavicular abduction. However, both structures lack sufficient power for correcting hindfoot valgus. Meanwhile, various medial ligaments were involved in AAFD, including the ligamentous supports of the talonavicular, subtalar, metatarsocuneiform, and naviculocuneiform joints [36]. A simple reconstruction of the spring ligament alone was insufficient to maintain the medial arch. In the CSPLR technique, the graft passed from the distal attachment of the peroneus longus to the anterior calcaneal hole functions similar to other plantar structures, which have a longer momentum arm compared to the spring ligaments to support the medial arch. Furthermore, in the CSILR technique, the graft extended from the hole in the navicular to the tibia could function as the tibionavicular ligament, which could restrain the talonavicular joint medial subluxation and forefoot abduction [49, 50]. Therefore, the importance of the superficial deltoid ligament, plantar fascia, and other plantar structures should be included in the reconstructive plan.

In our study, the spring ligament reconstruction corrected a simulated flatfoot deformity in the absence of procedures for other bony structures, such as those for calcaneal osteotomies and plantarflexion (Cotton) osteotomy of the medial cuneiform. This finding suggests that the spring ligament reconstruction mitigates the need for nonanatomic bony procedures associated with complications and allows for the preservation of the triple joint complex. However, we do not recommend performing spring ligament reconstruction for the correction of bony malalignment. The durability of these soft tissue reconstructive procedures, without the addition of bone realignment procedures, needs to be investigated further. AAFD presents in a wide variety of severities with differing ligament failures [36] and bony alignments [39, 51]. No one reconstruction technique is likely to correct the various deformities [5].

Despite the good qualitative and quantitative results that were obtained in this study, several limitations of our model must be noted. Firstly, our flatfoot model was developed based on a healthy foot. The simulation just evaluates a normal foot bone with various ligament reconstructions to mimic a flatfoot after surgery. Secondly, the loading model, which did not show the effect of the deformity on walking, was representative of standing. Thirdly, several simplifications were introduced in the modeling process. The parts of the graft pass thought the bone tunnels were simplified. We used material properties for soft tissues from the literature rather than actual measurements. Although attenuation values were applied to replicate the flatfoot condition, the exact stiffness values of each soft tissue structure were not known. Despite these limitations, our results were similar to the experimental measurements of previous studies.

## Conclusions

In conclusion, the nonanatomic reconstruction of the spring ligament complex provided the greatest degree of correction for midfoot and hindfoot misalignments in flatfoot. The reconstructive techniques that included the tibionavicular ligament or other plantar soft structures better corrected midfoot and hindfoot deformity in comparison to anatomic reconstruction. This work has provided a greater understanding of the biomechanical response of spring ligament reconstruction in AAFD. In future work, the efficacy of these procedures will be investigated in a substantial number of patients with a long-term follow-up period and optimal procedures for the patients will be developed.

## Abbreviations

AAFD: Adult-acquired flatfoot deformity
ASLR: Anatomic spring ligament reconstruction
CSILR: Combined superomedial/inferior ligament reconstruction
CSPLR: Combined superomedial/plantar ligament reconstruction
CT: Computerized tomography
FDL: Flexor digitorum longus
FE: Finite element
ICN: Inferior calcaneonavicular
ISLR: Inferior spring ligament reconstruction
MRI: Magnetic resonance imaging
SMCN: Superomedial calcaneonavicular
